# Fluid–Structure Interaction Analysis of a Bionic Robotic Fish Based on a Macrofiber Composite Material

**DOI:** 10.3390/biomimetics10060393

**Published:** 2025-06-11

**Authors:** Chenghong Zhang

**Affiliations:** School of Electronics & Information Engineering, Guiyang University, Guiyang 550005, China; zhangchenghongcn@aliyun.com

**Keywords:** fluid–structure interaction (FSI), macrofiber composite material (MFC), bionic robotic fish, turbulent model

## Abstract

In this study, the power system of a bionic robotic fish has been significantly simplified, resulting in a reduced volume and enhanced flexibility of both the structure and movement. To comprehensively understand the dynamics, a fluid–structure interaction (FSI) analysis was conducted, considering the intricate interplay between the mollusk’s structure and the surrounding fluid. This analysis took into account the dissipation due to fluid viscosity and the influence of the wake performance around the mollusk. The study examined the relationships between the driving frequency of the input signal and various parameters such as fluid pressure, propulsion force, and propulsion displacement of the soft robot fish head. With the robot fish’s head fixed, the amplitude of propulsion motion and propulsion force were measured. The simulation results closely matched the experimental findings, indicating their potential to predict the propulsion characteristics of the soft robot fish in fluid environments and further improve its performance.

## 1. Introduction

In nature, to adapt to different water environments, fish have evolved and use various swimming propulsion modes; this evolution process has become the focus of research in the fields of biology and bionics. In 1984, Webb classified the propulsion modes of fish into two types: body and/or caudal fin propulsion (BCF) and media and/or paired fin propulsion (MPF) [1,2]. The MPF mode is characterized by a slow propulsion speed and low efficiency and cannot meet the driving requirements of industrial underwater robots. Therefore, the current research on bionic robotic fish is mainly based on the BCF mode [3,4].

The BCF mode mainly uses fluctuations in the fish body or the swing of the caudal fin to push the water around the body diagonally backward; in this motion pattern, the reaction force of the water on the body yields forward thrust [5,6], which is further divided into wave propulsion and swing propulsion. Wave propulsion fish exhibit large lateral fluctuations, such as propulsion throughout their body during swimming, and can provide a complete waveform toward propulsion [7,8]. The swing propulsion fish body is divided into two parts: the matrix and the propulsion structure. The propulsion structure rotates around the matrix, and the whole body does not display a wave shape [9,10]. Generally, the main propulsion structure of swing propulsion fish is the caudal fin, and its propulsion efficiency is greater than that of body wave propulsion fish and is suitable for long-distance and long-term cruise swimming. However, wave propulsion fish tend to have greater flexibility and can adapt to more subtle and complex environmental changes.

As the drivers of most autonomous robotic fish, shape memory alloys provide a large driving force, but they also have the disadvantages of high energy dissipation and low efficiency. An ion-exchange polymer–metal composite (IPMC) is used as a driving element for microbionic fish, but the driving force is too small, and the effect is not ideal. Piezoelectric fiber composite materials (MFCs) are characterized by high energy conversion efficiency, a large driving force and good sealing ability, so they are suitable for driving bionic robotic fish.

In this study, we introduce a flexible bionic robotic fish utilizing an MFC and carbon-fiber-reinforced plastic (CFRP) to mimic the muscles and bones of fish tails, respectively. To elucidate the hydrodynamic propulsion capabilities of this robotic fish in fluid environments, it is imperative to conduct comprehensive hydrodynamic analyses, particularly considering the substantial deformation of the flexible robotic structure and its intricate coupling dynamics with the surrounding fluid. To this end, numerical models of both the soft robot structure and the ambient fluid are presented. Leveraging the MFC actuator drive model developed in ANSYS software ANSYS 15.0, a FSI analysis of the soft robotic fish is executed using computational fluid dynamics (CFD) methods. This analysis employs the MFC actuator drive model to assess the FSI of the robotic fish. Data pertaining to structural displacement and fluid pressure loads are exchanged at the interface between the robot structure and the fluid, facilitated by interface boundary conditions. Commencing with the coupled analysis, numerical simulations are conducted to evaluate the propulsion force and propulsion amplitude of the soft robotic fish in fluid. The relationships between the driving frequency of the robotic fish and both its propulsion force and displacement are meticulously examined. The hydrodynamic properties of the soft robotic fish are assessed, providing fundamental propulsion characteristics that can guide further improvements in the design and performance of these aquatic robots.

## 2. Introduction to Piezoelectric Intelligent Composite Materials

Piezoelectric fiber composites consist of piezoelectric fibers and resin matrices, meticulously combined in specific proportions, spatial distributions, and mutual connections. This unique composition leverages the strengths of both traditional piezoelectric polymers and resin matrices [11]. The resin matrix offers protective benefits, resulting in significant enhancements in the strength and brittleness of piezoelectric fiber composites compared to their polymer-only counterparts. Additionally, the flexibility and piezoelectric characteristics of these composite materials are vastly superior to those of traditional piezoelectric materials, primarily due to the optimized ratio and spatial distribution of the resin matrix [12].

MFC smart materials exhibit piezoelectric-controlled deformation capabilities, making them ideal for various applications requiring lightweight and high-efficiency actuators. In this research, an MFC intelligent piezoelectric actuator is chosen as the drive unit for a bionic robotic fish. By applying an external voltage, the deformation of the MFC driver can be precisely controlled, enabling the realization of a caudal fin swing propulsion mode. For this experiment, the M-8528-P1 MFC smart material manufactured by Smart Materials Corp. (Sarasota, FL, USA) was selected.

MFC integrate sensor performance and actuator performance; when these materials are used as actuators, the working principle is the inverse piezoelectric effect of the piezoelectric materials. An external voltage is applied to the electrodes of the MFC, creating strain at the working surface of the material. When the MFC is bonded to a CFRP substrate, the strain of the MFC acts on the substrate and causes it to deform because of the constraints of the substrate.

## 3. FSI Simulation of a Bionic Robotic Fish Based on MFC

### 3.1. Overview of the FSI Finite Element Analysis Method

In finite element analysis, the shape variable of a robotic fish structure is transferred to the fluid as a coupling variable, and the pressure of the fluid is transferred to the robotic fish structure as a coupling variable [13]. Through meshing, the original nonlinear structure is divided into multiple small linear units, each unit is connected, and an overall nonlinear calculation result is obtained; this approach solves the traditional nonlinear problem, which is normally difficult to solve [14].

### 3.2. Dynamics Theory of a Bionic Robotic Fish

**(1)**  **Structural Dynamics Model of a Bionic Robotic Fish**

**Figure 1:**
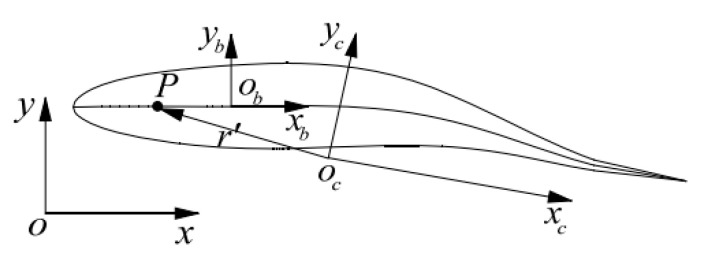
Robotic fish coordinate system model.

R. Wang proposed the three-coordinate system method for a fish-like robot [15,16]. In this method, the fish body coordinate system $o_{b}x_{b}y_{b}$ is established with the origin at the center of the robotic fish head. In addition, the center of mass coordinate system $o_{c}x_{c}y_{c}$ is established with the center of mass of the robotic fish as the origin, and the counterpart *oxy* is the inertial coordinate system. This coordinate system establishment method can decouple the external and internal forces of the robotic fish and is convenient to independently solve. The definition of the coordinate system is shown in Figure 1.

**(2)**  **Structural Dynamics Equations for the Bionic Robotic Fish**

In the process of motion, the bionic robotic fish is independently affected by the external force (moment) and the internal force (moment). The external forces (moments) influence the macroscopic motion of the bionic robotic fish in the inertial coordinate system, whereas the internal forces (moments) affect the deformation of the robotic fish itself [17]. The overall motion includes the translation and rotation of the robotic fish, and the resulting velocity includes the translational and rotational velocities of the center of the mass coordinate system. According to Newton’s classic mechanical theory, when only the external force and external moment are considered, the dynamic equation of the steady-state swimming motion of the bionic robotic fish can be obtained as follows:

(1) $$m{\dot{u}}_{c}\left(t\right)=F\left(t\right)$$

(2) $${\dot{I}}_{c}\left(t\right)\omega_{c}\left(t\right)+I_{c}\left(t\right){\dot{\omega }}_{c}\left(t\right)=M_{c}\left(t\right)$$

where *F*(*t*) is the external force of the robotic fish, *M_c_*(*t*) is the external moment of the robotic fish, *m* is the mass of the robotic fish, and *I_c_*(*t*) is the instantaneous moment of inertia of the robotic fish relative to the center of mass.

### 3.3. Theory of Fluid Dynamics

**(1)**  **Fluid Model**

**Figure 2:**
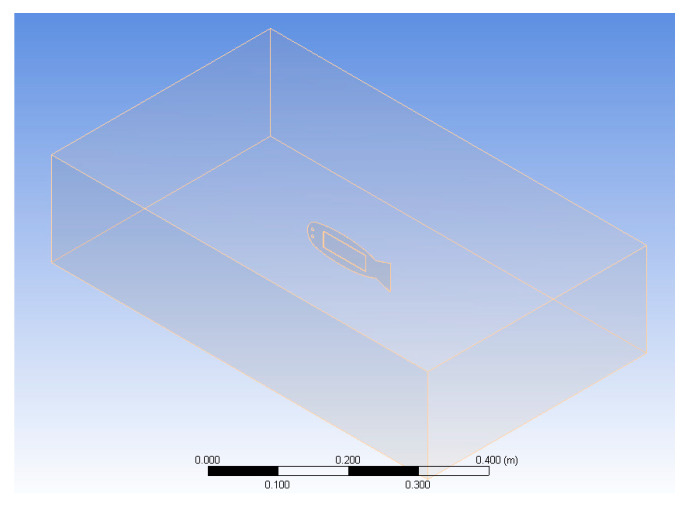
Fluid–structure interaction calculation domain.

As shown in Figure 2, the fluid computational domain in the FSI analysis is designed as a cuboid with a size of 590 mm× 133 mm× 440 mm, and the robotic fish structure model is centered in the fluid computational domain. Owing to the high-pressure driving characteristics of the MFC, in CFX analysis software ANSYS 15.0., the fluid material is set to FC-3283 (3M Corp, St. Paul, MN, USA), and the parameters are listed in Table 1. In addition, considering the actual experimental environment with a high pressure of 1500 V, the nontoxic, nonflammable and nonvolatile characteristics of fluorinated liquids can reduce the danger in experiments.

**(2)**  **Fluid Equations and Algorithm**

The Navier–Stokes equation is a motion equation that describes the conservation of momentum of viscous incompressible fluids and is by far the most complete system of fluid equations describing the motion of the flow field.

**Figure 3:**
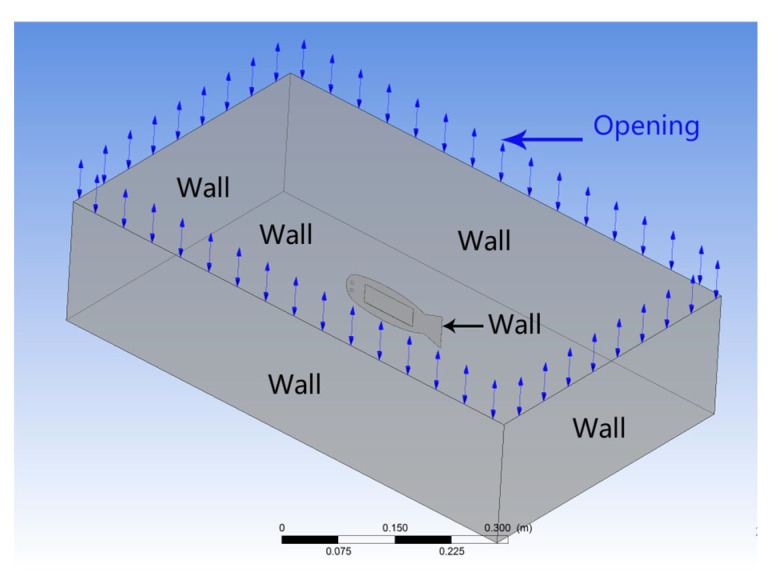
Fluid domain boundary conditions.

For incompressible viscous fluids [18], the following equations can be used:

(3) $$\rho \left(\frac{\partial V_{x}}{\partial t}+V_{x}\frac{\partial V_{x}}{\partial x}+V_{y}\frac{\partial V_{x}}{\partial y}+V_{z}\frac{\partial V_{x}}{\partial z}\right)=\mu \left(\frac{\partial^{2}V_{x}}{\partial x^{2}}+\frac{\partial^{2}V_{x}}{\partial y^{2}}+\frac{\partial^{2}V_{x}}{\partial z^{2}}\right)-\frac{\partial P}{\partial x}+F_{x}$$

(4) $$\rho \left(\frac{\partial V_{y}}{\partial t}+V_{x}\frac{\partial V_{y}}{\partial x}+V_{y}\frac{\partial V_{y}}{\partial y}+V_{z}\frac{\partial V_{y}}{\partial z}\right)=\mu \left(\frac{\partial^{2}V_{y}}{\partial x^{2}}+\frac{\partial^{2}V_{y}}{\partial y^{2}}+\frac{\partial^{2}V_{y}}{\partial z^{2}}\right)-\frac{\partial P}{\partial y}+F_{y}$$

(5) $$\rho \left(\frac{\partial V_{z}}{\partial t}+V_{x}\frac{\partial V_{z}}{\partial x}+V_{y}\frac{\partial V_{z}}{\partial y}+V_{z}\frac{\partial V_{z}}{\partial z}\right)=\mu \left(\frac{\partial^{2}V_{z}}{\partial x^{2}}+\frac{\partial^{2}V_{z}}{\partial y^{2}}+\frac{\partial^{2}V_{z}}{\partial z^{2}}\right)-\frac{\partial P}{\partial z}+F_{z}$$

(6) $$\nabla \cdot V=\frac{\partial V_{x}}{\partial x}+\frac{\partial V_{y}}{\partial y}+\frac{\partial V_{z}}{\partial z}=0$$

where *ρ* is the fluid density; *V* is the fluid velocity; and *V_x_*, *V_y_*, and *V_z_* are the components of the fluid velocity along the *x*-, *y*-, and *z*-axes, respectively. *P* is the pressure of the fluid, *μ* is the dynamic viscosity of the fluid, and *F_x_*, *F_y_*, and *F_z_* are the pressure components associated with the *x*-, *y*-, and *z*-axes, respectively. Owing to the large deformation effect and nonlinear characteristics of the robotic fish structure, the Navier–Stokes equation is used to calculate the fluid domain and can completely reflect the associated coupling characteristics. The fluid domain boundary conditions are shown in Figure 3. In the FSI simulation, two boundary conditions are applied to define the fluid model: opening and nonslip wall boundaries. The nonslip wall boundary condition is imposed on the surface of the entire robot body and the borders of the fluid domain, excluding the top surface. Under this condition, the fluid velocity at the wall boundary is set to zero, ensuring no slippage. Conversely, the top surface of the fluid domain is subjected to an opening boundary condition, where a pressure of zero is applied, allowing fluid to freely enter and exit this region. These boundary conditions effectively limit and manage the fluid dynamics within the simulation domain.

**(3)**  **Turbulence Model**

Turbulence is a nonlinear fluid motion that is spatially irregular and chaotic and has very complex flow patterns. Owing to its randomness, rotation, and dissipation, it is difficult to accurately simulate in fluid dynamics. In the fluid dynamics problem, the flow model is divided into laminar flow and turbulent flow. The laminar flow model is defined as follows: no interaction occurs between the adjacent layers of fluids, and only a relative tangential velocity exists between the fluids. However, the turbulence model has a normal velocity between the adjacent layers of fluids, fluid microgroups exhibit complex, irregular, and random unsteady motion, and macroscopic mixing occurs between the flow layers. This property in the flow model is described by the dimensionless Reynolds coefficient (*Re*). The Reynolds coefficient is low in the laminar flow state; here, the viscous force is the main force and is characterized by constant fluid motion. The Reynolds coefficient is high under turbulent conditions and is dominated by inertial forces. In a turbulent environment, unstable phenomena such as turbulence and whirlpools occur. The Reynolds coefficient is defined as follows [19]:

(7) $$R_{e}=\frac{\rho VL}{\mu }\;\mathrm{or}\;R_{e}=\frac{VL}{v}$$

where *ρ* is the fluid density, *V* is the average propulsion velocity, *μ* is the hydrodynamic viscosity, *v* is the fluid kinematic viscosity, and *L* is the length of the robotic fish.

According to Equation (7), the robotic fish at an average motion speed has a Reynolds coefficient that is greater than 10^4^, which is considered a high Reynolds coefficient. Therefore, the flow model is turbulent.

At present, in research on turbulence, the Reynolds averaging method is used to solve for variables, but the equations derived during the solution process introduce new variables, resulting in inaccurate results. Therefore, turbulence models are needed to determine these newly introduced unknown variables. Commonly used turbulence models include the semiequation model (Johnson–King model), two-equation model (*k*-*ε* and *k*-*ω* models), one-equation turbulence model (Spalart–Allmaras model), algebraic model (Baldwin–Lomax model), and Reynolds stress model [20].

In this study, the SST-type *k*-*ω* model is selected for FSI analysis; this model adds the deformation from the *k*-*ω* and *k*-*ε* models to the mixed-function dual model on the basis of the *k*-*ω* model. The cross-diffusion derived from the ω equation is combined with the resulting model. The *k*-*ω*-based shear stress transfer (SST) model is designed to provide high-precision prediction of flow separation under various pressure gradients by considering the transfer of turbulent shear stresses when calculating eddy current viscosity. The use of mixed functions to gradually transition from a standard *k*-*ω* model near the wall to a high Reynolds *k*-*ε* model outside the boundary layer is suitable for complex flow situations, especially those involving high shear stresses.

**(4)**  **Dynamic Mesh Model**

The dynamic mesh model in CFX is needed when solving problems with moving boundaries and a moving or deforming object within the computational area. The dynamic mesh model is special because the mesh distribution at the boundary of motion moves as the boundary moves. The boundary position at the next moment is determined according to the current boundary position, velocity, increment, and time increment, and the mesh in the area near the boundary is adjusted and modified. Areas with severe distortion are even rezoned to eliminate the mesh elements that are distorted because of boundary movement [21].

**Figure 4:**
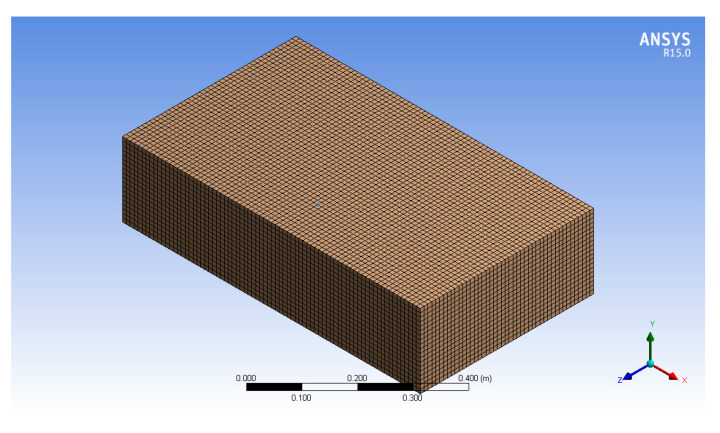
Fluid computation domain of the mesh model.

In the dynamic mesh model, the mesh properties are initially defined before calculations, and after a boundary is distorted or deformed, CFX automatically remeshes the calculation area. The equations of motion for boundary deformation are defined by boundary functions. The initial mesh model of the fluid calculation domain is shown in Figure 4.

### 3.4. Transient Analysis of the Bionic Robotic Fish

In this section, the FSI analysis method is used to conduct a transient analysis of a robotic fish structure and the surrounding fluid calculation domain, and the simulation model meets the following conditions: (1) the structure of the robotic fish is flexible and has a large deformation effect; (2) no flow occurs in the static fluid region; and (3) no heat exchange occurs in the environment.

Parameters are transferred on both sides of the interfaces of the fluid domain. Fluid pressure is applied to the structure as an external force, resulting in solid deformation. In addition, solid deformation generates fluid pressure at the interface of the fluid. The boundary conditions of the FSI are used to constrain the equations of motion at the interface. These boundary conditions include fluid continuity, velocity, pressure, and the normal and tangential displacement components at the interface, as shown in Equations (8) and (9). On the boundary, the fluid and solid domains have the same Cauchy stress tensor, as shown in Equations (10) and (11):

(8) $${v_{n}}^{s}={v_{n}}^{f}$$

(9) $${v_{\tau }}^{s}={v_{\tau }}^{f}$$

(10) $$\tau_{s}+\tau_{f}=0$$

(11) $$\sigma_{s}+\sigma_{f}=0$$

where *v_n_* and *v_τ_* are the normal and tangential velocities, respectively; *τ* and *σ* are the normal and shear stresses, respectively; and the superscripts *s* and *f* represent the solid and fluid calculation domains, respectively.

**Figure 5:**
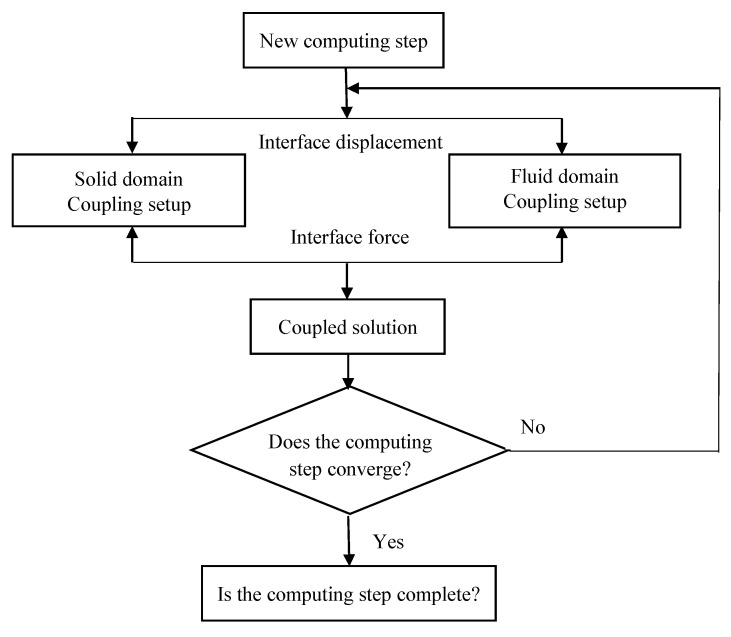
Flow chart of the FSI calculation process.

In transient analysis, the solid and fluid domains have distinct mesh and boundary conditions, and data exchange occurs at the interface. In each calculation step, the fluid domain solver uses input data and the results from the previous step in the solid domain, and the result is substituted into the next step of the calculation. The same process is applied for the solid domain solver. This calculation process is repeated until the data transfer and calculation domain equations for the studied interface converge, as shown in Figure 5.

To assess the hydrodynamic performance of the robotic fish, we must analyze the forward propulsion force as the fish moves. Since the length direction of the robotic fish is set as the *x*-axis and the caudal fin oscillates in the *z*-axis direction during modeling, we need to calculate the fluid pressure in the *x*-axis direction; this pressure can be obtained by integrating the fluid pressure and viscous pressure, as shown in Equation (12):

(12) $$F_{x}=\oint_{A}\left(-\vec{p}{\vec{n}}_{x}+{\vec{\tau }}_{xi}{\vec{n}}_{xi}\right)dA$$

where *dA* is the infinitesimal facial element of the robotic fish structure, ${\vec{n}}_{x}$ is the direction vector of facial element *dA* on the *x*-axis, and ${\vec{n}}_{i}$ is the *i*-th normal vector of facial element *dA*. $\vec{p}$ is the fluid pressure, and ${\vec{\tau }}_{xi}$ is the viscosity stress tensor associated with the *x*-axis.

## 4. Numerical Analysis of the Bionic Robotic Fish

The MFC is modeled as M-8528-P1 (Smart Materials Corp., USA), and the head weight is cylindrical structural steel with a diameter of 2.5 mm and a density of 7850 kg/m^3^. The thickness of the central CFRP plate is 0.2 mm, and the structural parameters of the other bionic robotic fish are listed in Table 2.

**Figure 6:**
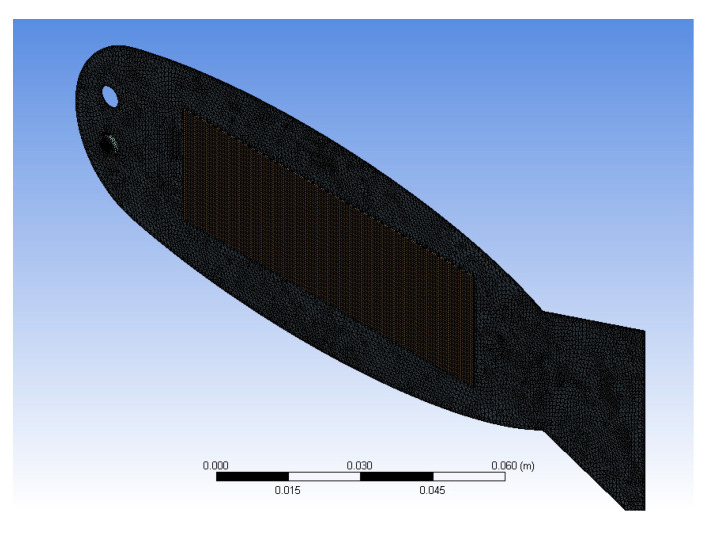
Finite element model of the bionic robotic fish.

The finite element model of the bionic robotic fish is shown in Figure 6.

### 4.1. Analysis of the Simulation Results of the Robot Swimming Pressure

**Figure 7:**
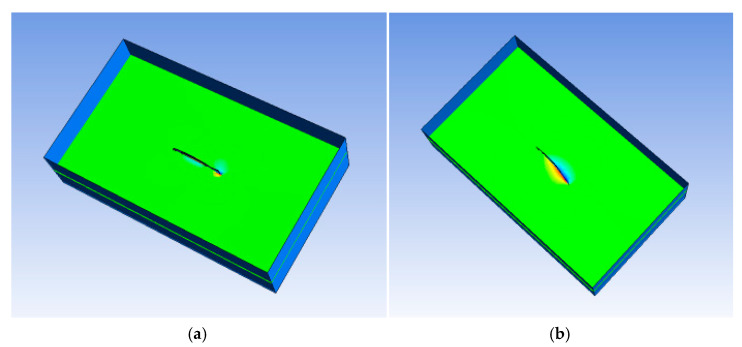

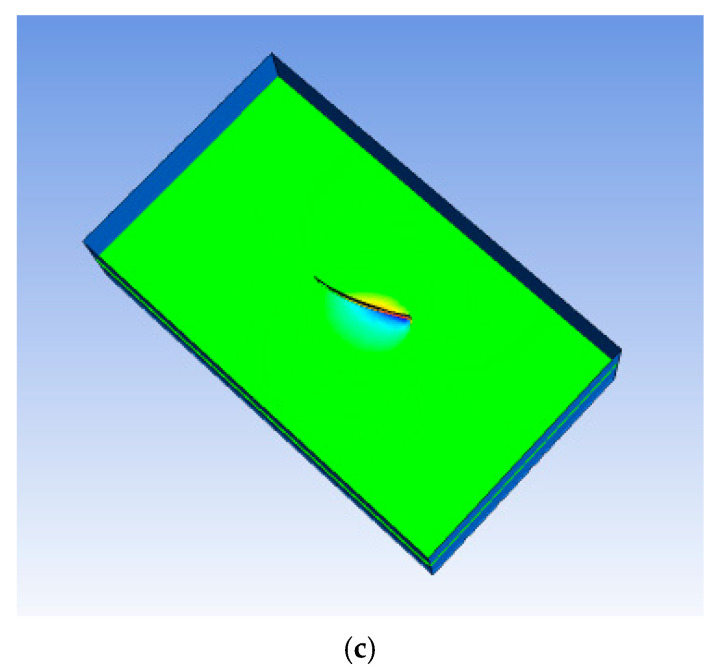
Pressure cloud diagram from the FSI analysis of bionic robotic fish. (**a**) Time = 0 s. (**b**) Time = 0.08 s. (**c**) Time = 0.25 s.

Fluid pressure plays a key role in the propulsion of soft fish robots. Depending on the specific actuation frequency of the robot, the pressure distribution around the robot changes over time. As part of the coupling of the interface, the fluid pressure plays a considerable role in the propulsion process of the bionic robotic fish. The frequency and amplitude of the driving signal vary, and the deformation effect of the obtained robotic fish is variable, resulting in different fluid pressures in the FSI analysis. Because the bionic robotic fish pushes the fluid backward by swinging its caudal fin, the reaction force of the fluid to the caudal fin is used to push forward. The pressure of the fluid domain around the robotic fish structure influences the magnitude of the corresponding reaction force, and this force directly affects the propulsion effect. Therefore, in this section, the pressure changes in the fluid domain are analyzed. On the basis of the number of swims *S*_w_ [22], the robot achieves effective flexible fish-like motion at 3 Hz, and the robot fish displays reasonable movements and responses. Figure 7 shows the fluid pressure cloud generated when a sine wave with a 3 Hz signal is used. In the bending and deformation of the robotic fish, the liquid flow near the structure of the robotic fish is considerably disturbed, and the fluid pressure is affected by the variable shape, velocity and velocity increment of the robotic fish.

As shown in Figure 7, the robot performs an oscillatory bending motion. The flow field pressure near the robot is considerably perturbed, and the dynamic pressure is influenced by the motion of the robot. The absolute value of the fluid pressure is the largest around the structure of the robotic fish. When the fish body is close to a domain boundary, the pressure is greatest. In the domain far from the structure, the pressure does not greatly change during the structural deformation process of the robotic fish. Owing to the oscillating bending motion of the robot, a considerable pressure difference exists between the left and right sides of the robot. In addition, the peak fluid pressure occurs close to the caudal fin of the robotic fish because the caudal deformation is the largest. On the basis of a comparison of the pressure clouds at different times, when the caudal fin reaches its peak shape, the pressure corresponding to the surrounding fluid calculation domain reaches the maximum value. Since the caudal fin of the robotic fish swings along the *z*-axis, the fluid pressure difference is large on both sides of the robotic fish along the *z*-axis direction. The final propulsion effect of the bionic robotic fish can be expressed as the sum of the fluid pressure in a cycle. When the pressure is high, a large driving force is generated, and the propulsion effect is best. Fluid pressure plays a key role in the propulsion of a soft fish robot. Depending on the specific actuation frequency of the robot, the pressure distribution around the robot changes over time.

### 4.2. Analysis of the Simulation Results of the Robot Fish Thrust and Displacement

**Figure 8:**
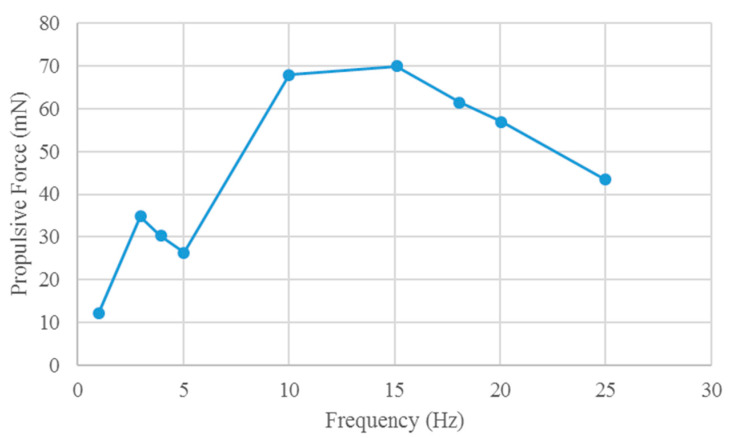
Propulsive force of the soft robotic fish in the simulation.

The hydrodynamic results along the direction of the robotic fish are shown in Figure 8. The input voltage amplitude range of the robot fish is −500~1500 V, and the frequency range is 1~25 Hz.

In Figure 8, the propulsion of the soft robotic fish with the robot head fixed at different drive frequencies is predicted via FSI analysis. The propulsion force differs among drive frequencies. No direct relationship exists between the propulsion force of a soft robot and the drive frequency.

**Figure 9:**
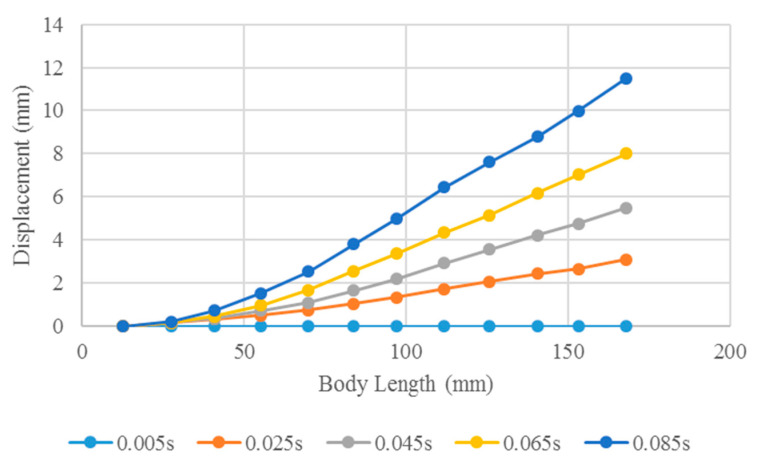
Deformation modes of the soft robotic fish in a quarter of one cycle at 3 Hz.

Through FSI analysis, the displacement of the soft robotic fish at different driving frequencies can be obtained. Soft robots perform oscillatory motions. At different drive frequencies, the soft robots used for propulsion exhibit similar bending patterns. The bending deformation mode of the robot is represented by good fish-like kinematic flexibility at 3 Hz, as shown in Figure 9. The results for a quarter of the period are described. As shown in Figure 9, the soft robot exhibits a bending deformation mode in oscillatory motion, and the displacement gradually increases from 0.005 to 0.085 s at 3 Hz. At 0.085 s, the deformation reaches a maximum. At different time points, the maximum deformation of the robot is observed at the end of the tail fin. Through FSI analysis, effective bending propulsion motion was verified for the soft robot.

**Figure 10:**
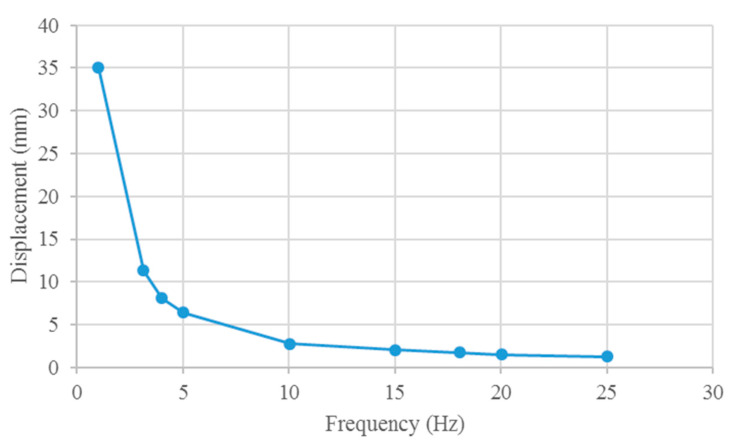
Displacement of the caudal fin end of the robot in the simulation.

The results of the tail fin tip displacement at different driving frequencies are shown in Figure 10. The maximum displacement at 1 Hz is approximately 34 mm. As the driving frequency of the soft robot increases, the displacement of the fish tail end decreases. By conducting FSI analysis, the relationship between the driving frequency and the displacement of the propulsion motion is determined, with the fish head of the soft robot fixed.

Through FSI simulation, the propulsion force and deformation trajectory of the soft robot fish are calculated at different driving frequencies. This analysis yields the relationship between the driving frequency of the input voltage and both the propulsion force and the displacement of the propulsion motion, with the head of the soft robot fixed in the fluid. FSI analysis serves as a valuable tool for predicting the propulsion performance of the soft robotic fish, thereby facilitating improvements in its overall performance.

## 5. Bionic Robotic Fish Experiment

**Figure 11:**
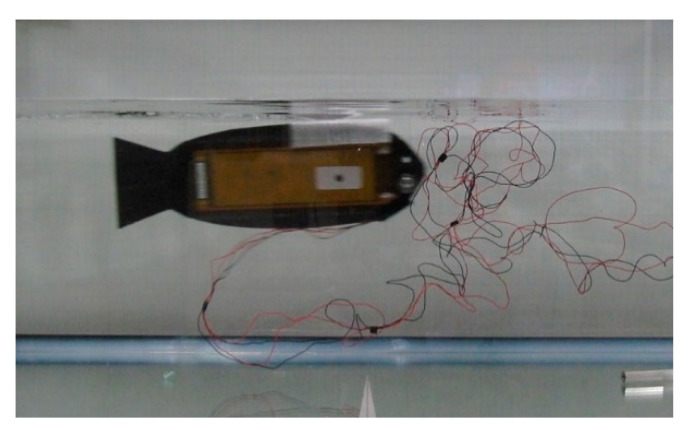
Bionic robotic fish.

To create an actuator structure capable of producing bending deformation for swimming movements, two MFC plates were clamped between CFRP plates. A steel weight was strategically placed on the head of the robot to enhance the displacement of the tail end. To ensure stability of the prototype within the fluid, a low-density blowing agent was utilized as a float and positioned on top of the prototype, effectively balancing its body weight. The bionic robotic fish is shown in Figure 11.

### 5.1. Propulsion of a Molluskular Robot

**Figure 12:**
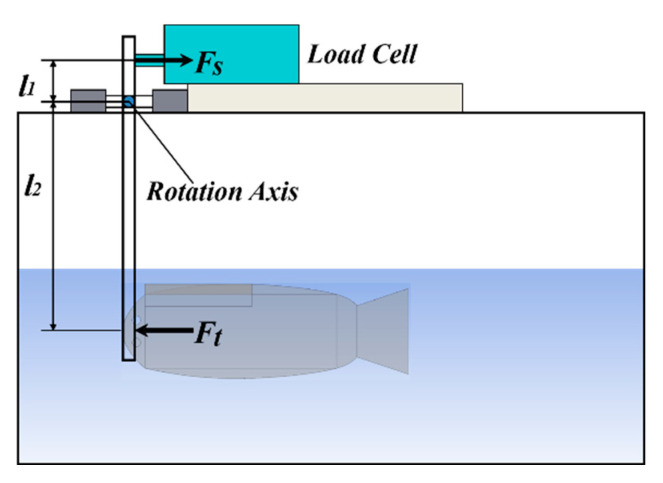
Experimental platform of the propulsive force for the prototype.

**Figure 13:**
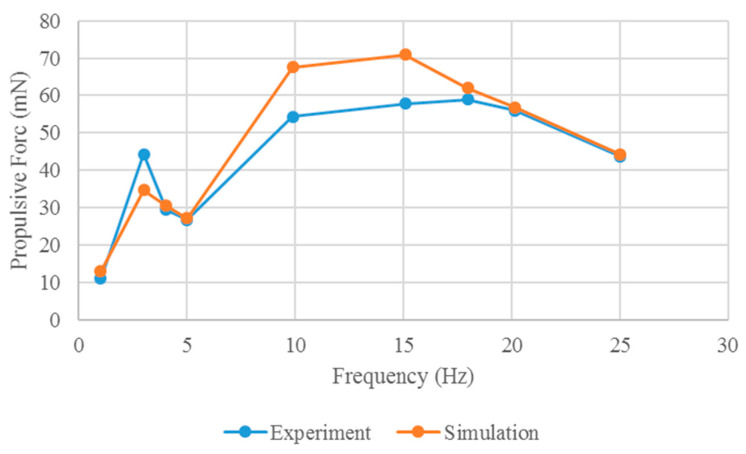
Propulsive force of the prototype in the experiment.

In the soft robotic fish experimental platform, a high-speed camera was used for prototype measurements. The platform for measuring the propulsive force of the prototype comprises a force gauge (single-axis load cell) and a stick. It is mounted on the upper part of the stick, which rotates around a fixed axis. The lower part of the stick makes contact with the head of the robot prototype. As input voltage is applied to the robot, it generates propulsion force, causing the stick to rotate around the axis. This rotational motion transfers the propulsion force to the upper part of the stick, where it is registered by the load cell. The experimental platform for determining the propulsive force of the prototype is shown in Figure 12. The propulsion forces of the prototype at different drive frequencies are shown in Figure 13.

As illustrated in Figure 13, no direct correlation exists between the propulsion force and the drive frequency of the robot prototype. Comparison of the prototype experiments with the simulation results of the FSI analysis reveals a similar trend in propulsion. However, owing to the fluid model and boundary conditions employed in the simulation, notable discrepancies in force are observed within the frequency range of 10~18 Hz. In the simulation, a numerical fluid model is utilized to approximate the turbulent physical properties of the FSI solution, where the loads exerted by fluids on the robot structure are approximated, leading to errors between the simulated and measured results. For the numerical analysis, a homogeneous fluid model and boundary conditions were selected. Despite these deviations, the simulation results closely resemble the experimental outcomes, successfully capturing the trend of the propulsion force of the mollusk robot at varying frequencies.

### 5.2. Displacement of the Soft Robotic Fish

**Figure 14:**
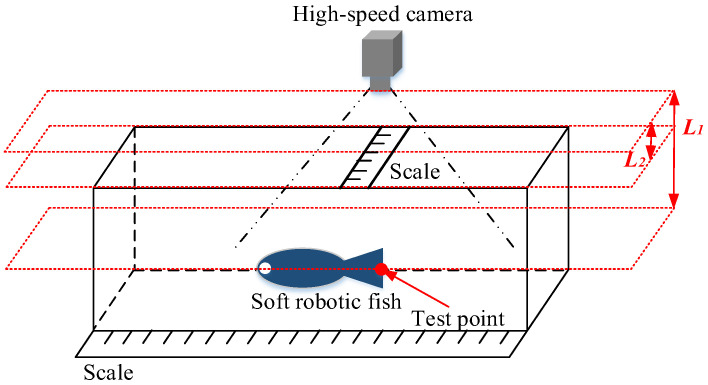
Experimental platform and test points at the end of the tail.

**Figure 15:**
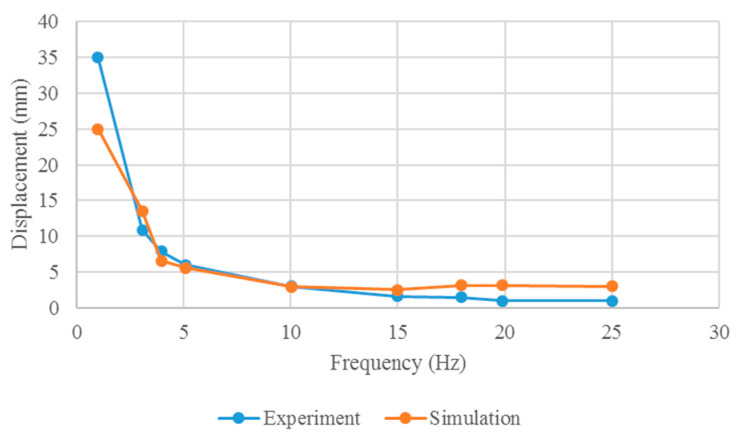
Displacement at the caudal fin end of the prototype.

To determine the oscillating amplitude of the prototype, a scale is positioned atop the fluid tank. The distances *L*_1_ and *L*_2_ are meticulously measured, where *L*_1_ is the distance between the test point on the prototype and the camera, and *L*_2_ is the distance between the scale and the camera. Utilizing the ratio between *L*_1_ and *L*_2_, the actual oscillating amplitude of the test point is accurately calculated. The experimental platform and test points on the prototype are shown in Figure 14. The displacement at the caudal fin end of the prototype is shown in Figure 15.

From Figure 15, similar displacement curves are evident in both simulations and experiments. Within a specific range, the tail displacement decreases as the drive frequency increases. Notably, the maximum displacement in both simulations and experiments occurs at a frequency of 1 Hz, which does not correspond to the frequency that yields the maximum propulsion. This phenomenon can be attributed to the fluid absorbing the energy generated by resonance, preventing the full transfer of resonance energy to the robot structure. At 1 Hz, the maximum simulated tail deformation of the robot is approximately 34 mm, whereas the maximum experimental tail deformation is approximately 24.7 mm, resulting in a maximum displacement difference of approximately 39%. The smallest difference in displacement, approximately 5%, occurs at a frequency of 10 Hz. Overall, the simulation results for tail displacement closely match the experimental results for the prototype, demonstrating that FSI analysis can effectively predict the deformation characteristics of robotic mollusks.

Using the actual bionic robot, the amplitude of the propulsion motion and the vortex distribution around the robot fish were measured with the robot head fixed. The numerical simulation results were basically consistent with the experimental results. The effectiveness of the modeling method and numerical coupling analysis was verified, indicating that these methods can be used to predict the propulsion characteristics of soft robotic fish in fluids to improve their performance.

## 6. Conclusions

In this work, an MFC was used as a soft actuator to develop a bionic soft robotic fish. The finite element method was used to model the MFC piezoelectric composites and CFRP substrates, and a finite element model of the piezoelectric cantilever beam was established after the corresponding material models were obtained. The FSI simulation of the bionic robot fish was performed on the basis of the dynamic and fluid models of the bionic robot fish. Here, the structural model of the bionic robotic fish was based on the three-coordinate system method. Through different actions, the internal force deforms the body of the robot fish, and the external force (moment) causes the robot fish to swing its tail fin. The dynamic meshing method was applied in an FSI analysis. Each calculation step involves calculation data and displacement from previous steps, and the mesh is redivided according to the previous structural displacement at the interface to ensure the quality of the mesh and the accuracy of the calculation results in subsequent steps.

The coupled problem of a soft robotic fish in a surrounding fluid was solved by modeling the interaction between the flexible robot structure and the fluid. FSI analysis was performed on the soft robot fish given the interaction of the soft robot structure with the surrounding fluid. Through FSI analysis, the hydrodynamic forces and displacements of a molluskular robot at different driving frequencies were calculated. The hydrodynamic properties and displacement of the mollusk robot were successfully assessed. Similar propulsion and displacement curves were observed in the simulations and experiments at different driving frequencies. Within a certain range, displacement decreases with increasing driving frequency of the soft robotic fish. FSI analysis can be used to characterize the hydrodynamic performance of molluskular and fish robots and evaluate their propulsion behavior in fluids.

## Figures and Tables

**Table 1:** Fluid domain parameters.

Item	Parameter Value
Density (kg/m^3^)	1820
Molar mass (g/mol)	521
Kinematic viscosity (cSt)	0.75
Absolute viscosity (cSt)	1.4

**Table 2:** Structural parameters of the robotic fish model.

Item	Parameter
Body length (mm)	167
Maximum body height (mm)	55
Caudal fin height (mm)	50
Body thickness (mm)	CFRP 0.2
Actuator type	M-8528-P1
Actuator size (mm)	112 × 40
Active area size (mm)	85 × 28

## Data Availability

The original contributions presented in this study are included in the article.
